# Treatment of nicotine dependence needs be strengthened in primary health care

**DOI:** 10.1080/02813432.2022.2159638

**Published:** 2023-01-02

**Authors:** Mikael O. Ekblad

**Affiliations:** a Department of General Practice, University of Turku and Turku University Hospital, Turku, Finland; b National editor of Scandinavian Journal of Primary Health Care

Smoking is a major cause of morbidity and mortality. Smoking causes, e.g. chronic obstructive pulmonary disease (COPD), pulmonary cancer, cardiovascular diseases, and increases the risk for postoperative complications. The prevalence of smoking has significantly decreased in the Nordic Countries during the last decades. According to the European health interview survey, the prevalence of daily smoking is between 6 and 12% in the Nordic Countries [1]. However, the prevalence of smoking remains especially high among certain populations, including but not limited to pregnant teenagers, patients with psychiatric problems, and even primary health care patients with depressive symptoms without clinical depression compared to non-depressed population [2]. It is known that smokers utilize primary health care services more often already before the inevitable diagnosis of smoking-related illnesses [3]. Thus, smoking causes an economic burden on the health care system. More effective treatment of nicotine dependence in primary health care would save lives and money.

Smoking cessation is the best way to decelerate the progression and to reduce the mortality of COPD. There are several studies showing that COPD patients are regularly educated regarding the effects of smoking and encouraged to quit smoking by healthcare professionals in primary health care [4,5]. Molin et al. [5] examined the extent of smoking cessation in COPD patients from the perspective of general practitioners (GPs) in Denmark (*n* = 470). All GPs reported that they discuss about the importance of smoking cessation with COPD patients. However, only 58% of the respondents were more likely to define specific targets for smoking cessation and 35% planned future consultations. Sandelowsky et al. [6] investigated the self-reported needs for information about COPD in primary health care patients with either moderate or severe COPD (*n* = 542) in Sweden. Despite the patients reported the least need for information about medicines and smoking, the authors found alarmingly that almost 60% of the patients who were current smokers or recent quitters, had not been offered smoking cessation support, and 80% had not previously been given such support by their GPs. These studies emphasize from the perspective of both the GPs and patients that there is a need for more effective treatment for nicotine dependence in primary health care.

The evidence of the efficacy and safety of both pharmacological and behavioural interventions for treating nicotine dependence is robust [7,8] and these should be exploited more effectively. Another example of this need is a real-world study by Gräsbeck et al. [9] on primary health care referrals of all patients aged 16 and over who underwent surgery at Porvoo Hospital in Finland (*n* = 1482). They found a weak smoking cessation awareness in primary health care before surgery, as preoperative smoking cessation for current smokers was visible in only 2.2% of referrals.

A new challenge is the emergence of diverse new nicotine-containing products that maintain nicotine dependency and attract new users. It is important to bear in mind that the use of nicotine in its various forms (smoking, snuff, e-cigarettes, nicotine bags, etc.) remains still very common despite the decreasing trend of traditional smoking. Nicotine is known to be responsible for many of the harms caused by smoking. One way to emphasize the importance of nicotine dependency to the patient would be to use more actively the International Classification of Diseases 10 -code (ICD-10) F17.29 for nicotine dependence. A qualitative study by Lönnberg et al. [10], including participants with a high risk of cardiovascular disease, implied that lifestyle change is a personal matter, but the support provided by health care personnel is essential to increase motivation and self-efficacy to change lifestyles. Thus, the GPs have a key role in encouraging patients to have healthier lifestyle including quitting smoking and use of other nicotine products.

In conclusion, it is essential to comprehensively assess the use of different nicotine products by the patient. The target should not only be to quit smoking but to stop the use of all nicotine products. There is undoubtedly a need to strengthen the treatment of nicotine dependence in primary health care. This could be achieved for example by developing local treatment chains for the treatment of nicotine dependence and by utilizing more often national online cessation programs. The emergence of new nicotine products emphasizes the importance of tackling the use of not only smoking but also all other nicotine products in primary health care.
